# DNA Damage, Repair, and Cancer Metabolism

**DOI:** 10.3390/ijms242216430

**Published:** 2023-11-17

**Authors:** Tae-Hong Kang

**Affiliations:** Department of Biomedical Sciences, Dong-A University, Busan 49315, Republic of Korea; thkang@dau.ac.kr; Tel.: +82-51-200-7261

The intricate interplay between DNA damage response (DDR) and metabolism unveils a profound insight into the fundamental mechanisms governing the maintenance of genomic integrity [1]. Cells constantly encounter endogenous and exogenous threats that induce DNA damage, which, if left unrepaired, can lead to mutations, genomic instability, and ultimately, diseases such as cancer [2]. Metabolism, on the other hand, provides the necessary energy and building blocks for DNA repair processes [3]. Notably, key signaling pathways and enzymatic activities in both DDR and metabolism are tightly interconnected. For instance, the activation of the ATM and ATR kinases in response to DNA damage directly influences the cellular metabolic state by regulating the mTOR pathway and cellular energetics [4]. Moreover, DNA repair enzymes, such as PARP1, are intricately linked to NAD+ metabolism, and their activities impact cellular bioenergetics [5]. This intricate crosstalk between DDR and metabolism not only ensures genome stability but also underscores the vital role of cellular homeostasis in safeguarding genetic information, making it a critical area of study with far-reaching implications for human health and disease.

This Special Issue presents a collection of nine papers from leading experts in the fields of DNA damage response and cancer metabolism. These papers highlight the latest advances in pharmacokinetic and pharmacodynamic analyses of specific DNA-damaging agents and the discovery of novel factors and regulatory mechanisms within the DDR, including DNA repair, checkpoint pathways, replication stress, cell death, and cancer metabolism. Additionally, these papers shed light on the intricate crosstalk between these systems, providing valuable insights into the broader landscape of genomic stability and cellular metabolism against DNA damage.

Park et al. delve into the role of the zinc finger protein ZATT in the context of Etoposide (ETO) treatment, revealing its dual functions in the repair of the topoisomerase II (TOP2)-DNA covalent complex (TOP2cc) and promoting cell survival following ETO treatment. ETO stabilizes the transient TOP2cc, resulting in DNA double-strand breaks (DSBs). The repair of TOP2cc involves tyrosyl-DNA phosphodiesterase 2 (TDP2), which removes phosphotyrosyl peptides from the 5′-termini of DSBs. This study employed genome-wide CRISPR screens and demonstrated that ZATT plays a crucial role in promoting cell survival following ETO treatment, with ZATT knockout (KO) cells showing heightened sensitivity to ETO compared to TDP2-KO cells. Further investigations into ZATT’s structural aspects revealed that the N-terminal 1-168 residues are essential for its interaction with TOP2, significantly influencing ETO sensitivity. Depletion of ZATT was found to accelerate TOP2 degradation after ETO or cycloheximide treatment, indicating its role in increasing TOP2 stability and likely contributing to TOP2 turnover. These findings suggest that ZATT is a critical determinant in the response to ETO treatment, holding promise as a strategy to enhance ETO’s efficacy in cancer therapy.

Yeom et al. investigate the functional properties of three human POLH germline variants associated with DNA polymerase η, a critical enzyme responsible for error-free translesion DNA synthesis (TLS). These variants are linked to the skin cancer-prone condition (i.e., xeroderma pigmentosum variant (XPV)) and increased sensitivity to cisplatin. Biochemical and cell-based assays were utilized to assess the impacts of these germline variants. In enzymatic assays, three variants (C34W, I147N, and R167Q) exhibited significantly decreased specificity constants for dATP insertion opposite specific DNA lesions compared to the wild-type enzyme, while others showed increased activity. A POLH knockout increased sensitivity to UV and cisplatin in human embryonic kidney cells, and this sensitivity was reversed by ectopic expression of wild-type POLH. However, the C34W, I147N, and R167Q variants, which exhibited substantial reductions in TLS activity, were unable to rescue the UV- and cisplatin-sensitivity, raising the possibility that these hypoactive germline POLH variants may heighten susceptibility to UV irradiation and cisplatin chemotherapy.

The research from Han et al. focuses on the transcriptional silencing of CD24, CD44, CD133, and CD147, which are recognized as cancer stem cell surface markers in various cancer types, including oral squamous cell carcinoma (OSCC). Through examination of several OSCC cell lines, the study establishes a clear correlation between the transcriptional expression levels of these genes and their reactivation by 5-aza-2′-deoxycytidine (5-aza-dC), as well as their promoter methylation status. Notably, the CD24, CD133, and CD147 genes exhibit promoter hypermethylation at significantly higher levels (70% and 75%, respectively) in OSCC cell lines compared to normal oral mucosa tissues (<53%), underscoring the cancer-specific nature of this methylation pattern. Moreover, the study extends its findings by analyzing the expression and methylation profiles of CD133 and CD147 in OSCC tumors obtained from The Cancer Genome Atlas (TCGA) database, revealing a negative correlation that supports their epigenetic regulation in primary OSCC tumors. Importantly, the methylation status of CD133 and CD147 is associated with poor survival in OSCC patients, as evidenced by the TCGA database. These findings provide valuable insights into the abnormal DNA methylation of CD133 and suggest the potential utility of CD147 as a diagnostic and therapeutic target for individuals with OSCC.

The review from Shin and Lee highlights the emergence of the zebrafish as a powerful model organism for investigating DNA repair mechanisms, benefiting from its well-characterized genomic information, the ability to visualize specific phenotypic outcomes in different cells and tissues, and the availability of diverse genetic experimental approaches. This review provides an in-depth overview of recent advancements in understanding the in vivo roles of DNA repair pathways, covering critical biological processes such as neurogenesis, hematopoiesis, germ cell development, tumorigenesis, and aging. With a specific emphasis on findings obtained from zebrafish research, this comprehensive review underscores the significance of using zebrafish as a model system to enhance our comprehension of DNA repair system functions at the organismal level, paving the way for future investigations in this field.

The ubiquitin-proteasome system (UPS) and E3 ligases, such as NEDD4, APC/CCDH1, FBXW7, and Pellino1, bridge the gap between cellular metabolism and DDR, making E3 ligases with high substrate specificity potential therapeutic targets. Numerous small-molecule inhibitors targeting UPS components have been developed and tested in clinical trials, offering promise for cancer treatment. In the review paper entitled “Ubiquitin Links DNA Damage and Repair Signaling to Cancer Metabolism”, Koo et al. provide insights into the role of ubiquitination in cellular metabolism and DDR, emphasizing the connections between these processes. The review also offers an overview of clinically significant small-molecule inhibitors, highlighting their potential practical use in cancer therapy.

The review from Lee et al. highlights the significant roles of genetic mutations and environmental agents in leukemia development, primarily attributed to genomic instability. It delves into the concept of R-loops, three-stranded nucleic acid structures composed of an RNA-DNA hybrid and a non-template single-stranded DNA, which have a crucial role in regulating cellular processes, including transcription, replication, and DNA double-strand break repair. However, when R-loop formation becomes unregulated, it can lead to DNA damage and genomic instability, potentially contributing to cancer, particularly leukemia. The review also explores the potential of targeting R-loops as a therapeutic approach in cancer treatment, shedding light on novel strategies to combat leukemia.

The review from Moon et al. underscores the dual role of DNA damage in cancer cells, as it can both increase the risk of mutations and tumorigenesis while also serving as an effective means to kill cancer cells when induced by chemical reagents or radiation. It highlights that mutations in key DNA repair genes, such as BRCA1 and BRCA2, can lead to genomic instability and promote cancer development, making cancer cells with these mutations more sensitive to chemotherapy or radiotherapy due to reduced DNA repair efficiency. The study also suggests that targeting key enzymes in the DNA repair pathway with specific inhibitors could induce synthetic lethality with chemotherapy or radiotherapy, providing a potential strategy for cancer therapeutics.

The review from Kim and Yoon emphasizes the pivotal role of gamma-aminobutyric acid (GABA) in signal transduction and neurotransmission and discusses the need for a deeper understanding of its cellular functions and physiological relevance in metabolic organs beyond the brain. The focus is on GABA metabolism, specifically its biosynthesis and functions in the liver, shedding light on how GABA synthesis is linked to cellular function in these organs. The review underscores the importance of investigating the effects of GABA and GABA-mediated metabolites in physiological pathways, with potential implications for mitigating metabolic diseases, and calls for further research into the multifaceted effects of GABA on metabolic disease progression.

Sohn et al. review importance of vision for daily life and the prevalence of eye diseases such as cataracts, diabetic retinopathy (DR), age-related macular degeneration (ARMD), and glaucoma, which can lead to blindness as individuals age. It highlights the successful outcomes of cataract surgery in the absence of additional visual pathway pathologies but points out the challenges faced by patients with DR, ARMD, and glaucoma, which often result in significant visual impairment. The review also introduces the role of DNA damage and repair as significant factors in the development of these eye diseases, underscoring the genetic and hereditary components involved in their pathogenesis and offering valuable insights into potential mechanisms for their prevention and treatment.

The collection of papers presented in this Special Issue highlights the significance of genomic integrity and the DNA damage response (DDR) in maintaining overall health and the challenges posed by various DNA-damaging agents. The DDR, with its intricate mechanisms involving DNA repair, cell cycle regulation, replication stress responses, and programmed cell death, plays a pivotal role in safeguarding cells from the constant threats to genomic stability. A deeper understanding of the DDR promises new avenues for preventing and treating conditions resulting from DNA damage, including the complex challenges of cancer and aging. The individual studies provide valuable insights into specific aspects of the DDR, shedding light on the dual functions of proteins like ZATT, the impact of POLH germline variants, epigenetic regulation of cancer stem cell markers, the significance of DNA repair in zebrafish, the potential of targeting ubiquitination in cancer therapy, the role of R-loops in genomic instability and leukemia, and the interplay between DNA damage, DNA repair, and metabolic diseases in various organs.

These findings collectively contribute to the broader landscape of understanding genomic stability and cellular defense mechanisms. Importantly, they provide new avenues for the development of preventive, diagnostic, and therapeutic strategies. The potential therapeutic targets and insights offered by these studies hold promise in the ongoing efforts to combat cancer and age-related diseases driven by genomic instability. The combination of these perspectives adds to the growing body of knowledge, further advancing our understanding of DNA damage and repair pathways and their implications for human health and disease. This comprehensive exploration of the DNA damage response is a testament to the importance of continued research in this critical field, emphasizing the potential for innovative approaches to promote healthy aging and disease prevention.

As the Guest Editor, I wish to express my heartfelt gratitude to all the authors for their outstanding contributions and to the Editorial Board for their unwavering professional support.
